# Clozapine: a review of clinical practice guidelines and prescribing trends

**DOI:** 10.1186/1471-244X-14-102

**Published:** 2014-04-07

**Authors:** Stephanie Warnez, Silvia Alessi-Severini

**Affiliations:** 1 Faculty of Pharmacy, University of Manitoba, Winnipeg, MB, Canada

**Keywords:** Clozapine, Prescribing, Schizophrenia, Antipsychotic(s), Utilization, Guidelines

## Abstract

**Background:**

Clozapine effectiveness in the treatment of refractory schizophrenia has been sustained by published evidence in the last two decades, despite the introduction of safer options.

**Discussion:**

Current clinical practice guidelines have strongly recommended the use of clozapine in treatment-resistant schizophrenia, but prescribing trends do not appear to have followed such recommendations. Clozapine is still underutilized especially in patients at risk of suicide. It seems that physicians are hesitant in prescribing clozapine due to concerns about serious adverse effects. Recent reports have highlighted the need to inform health professionals about the benefits of treating patients with clozapine and have voiced concerns about the underutilization of clozapine especially in patients at risk of suicide.

**Summary:**

Guidelines and prescribing patterns reported in various countries worldwide are discussed. Suggestions on how to optimize clozapine utilization have been published but more efforts are needed to properly inform and support prescribers’ practices.

## Background

Clozapine efficacy as an “atypical” antipsychotic agent has been recognized since the early 1960s [1]. This agent became available on the European market for the treatment of schizophrenia with the promise of a better tolerability because of the absence of those extrapyramidal side effects that afflicted patients treated with other antipsychotic agents (e.g., chlorpromazine and haloperidol, now defined as “typical” or first- generation agents, FGAs) [1]. However, the release of alarming reports of agranulocytosis in Finnish patients created panic among prescribers; a total of 17 cases were confirmed among 100 patients treated with clozapine, 8 of those cases resulted in fatalities. Clozapine was immediately withdrawn from the market by Sandoz (1976) [1].

It was not until fourteen years after its withdrawal that the results of the pivotal US Clozaril Study were published and the important role of clozapine in clinical practice was discovered [2]. Clozapine demonstrated superiority to chlorpromazine in treatment-resistant patients in many outcomes including treatment response, with improvements in positive and negative symptoms of schizophrenia [2]. This led health agencies to grant clozapine access to the US and Canadian markets in 1990 and 1991, respectively.

The recognized risk for agranulocytosis, assessed at a prevalence of 1-2%, has been managed since then by mandatory monitoring systems administered by the manufacturers of the clozapine products (now generized in most countries) to assure that patients undergo routine blood testing before each dispensation. Current product monographs mandate that patients start therapy gradually and that normal WBC and absolute neutrophil counts (ANC) are maintained at safe levels (WBC count ≥3500/mm^3^ and ANC ≥2000/mm^3^) throughout treatment [3]. Thus, patients at risk of developing agranulocytosis can be identified before the condition becomes life-threatening. The implementation of registries for the monitoring of hematological toxicity has in fact significantly reduced mortality and morbidity in patients treated with clozapine [4].

The 1990s saw also the introduction of other new “atypical” antipsychotics (e.g., risperidone, olanzapine, quetiapine also identified as second-generation agents, SGAs). These agents, developed to obtain efficacious medications similar to clozapine without the hematological toxicity, generated hope for improved compliance and Quality of Life (QoL) in patients affected by schizophrenia. Early studies of SGAs demonstrated superiority over the FGAs, including a lower incidence of EPS, superior efficacy for positive, negative and mood symptoms, improved tolerability, as well as cognitive enhancing effects [5-8]. Despite higher costs, SGAs were widely adopted worldwide [9-12]. However, none of the newer SGAs could demonstrate superiority to clozapine and in the last decade many published clinical practice guidelines [13-20] have recommended that clozapine be prescribed to patients with treatment-resistant schizophrenia, which has been defined as not responsive to two trials of any other antipsychotic medication (either FGA or SGA). Despite such recommendations and the overwhelming evidence of clozapine effectiveness, prescribing of clozapine appears to be low, delayed and often preceded by attempts at polypharmacy treatment, which lacks clinical evidence of effectiveness [21-26].

### Aim and scope

The main objectives of this article are to provide information on clozapine place in therapy and to discuss clozapine’s prescribing trends since the publication of the most recent schizophrenia clinical practice guidelines in North America and Europe.

## Methods

Clinical practice guidelines, meta-analyses, and reports on clozapine prescribing trends have been identified from searches in PubMed, Psychinfo, EMBASE and Cochrane databases. The search was focused on publications in the last 10-year period (2004 and 2014). Articles selection was limited to those written in the English language. Individual clinical trials and older landmark studies have been cited where appropriate. Keywords used in the literature search included: clozapine, prescribing, schizophrenia, antipsychotic(s), utilization, guidelines.

## Results

### Clozapine vs. FGAs

Clozapine effectiveness was initially demonstrated against chlorpromazine, the prototype of the FGAs [2]. Evidence of clozapine advantage over FGAs has been well established and results of various trials, conducted mostly in the 1990s, have been included in meta-analyses [27-29]. The conclusions were that clozapine was more effective than FGAs in reducing symptoms of schizophrenia according to measurements of BPRS scores and SANS negative symptoms scores. Clozapine produced clinically meaningful improvements and postponed relapse [30-32].

### Clozapine vs. SGAs

The landmark study CUtLASS 2 found that in patients failing to respond to two or more antipsychotics, clozapine produced significant improvement in symptoms over 1 year [33]. The phase 2E CATIE study revealed that clozapine was significantly more effective than quetiapine and risperidone, and the authors concluded that in patients with an insufficient therapeutic response to a SGA, clozapine was more effective than switching to a different SGA [34-36].

More recent evidence has confirmed clozapine superiority to other SGAs and olanzapine [37,38].

### Clozapine effectiveness on mortality

Effects of clozapine treatment on patient mortality has been assessed and reviewed over the years in various populations. The evidence supports a clear advantage of clozapine therapy in terms of reduced mortality over other antipsychotic agents including FGAs, risperidone and quetiapine [39-42].

### Clinical practice guidelines and algorithms

The evidence produced in the last 20 years, and summarized by published clinical practice guidelines and algorithms, has confirmed clozapine to be the gold standard for treatment-resistant schizophrenia. Please refer to Table 1 for a summary of recommendations.

**Table 1:** Summary of evidence-based recommendations for clozapine prescribing

**Date**	**Source**	**Recommendation: clozapine to be prescribed or offered to patients**		
		**After failure of 2 adequate trials of 2 different AAs**	**With suicidal thoughts or behaviours**	**With persistent hostility and violent behaviours**
2004	APA (US) [13]	**X**		
2005	CPA (CAN) [14]	**X**		
2007	TMAP (US) [15]	**X** (SGAs considered first-line)		
2009	NICE (UK) [16]	**X** (at least one of the drugs should be a non-clozapine SGA)		
2010	Schizophrenia PORT (US) [17]	**X**	**X**	**X**
2010	CADTH [18]	**X**		
2011	BAP (UK) [19]	**X**		**X**
2013	PAP (US) [20]	**X** (SGA, risperidone and olanzapine) considered first-line)	**X**	**X**

Note: AA = antipsychotic agent, APA = American Psychiatric Association, BAP = British Association for Psychopharmacology, CADTH = Canadian Agency for Drugs and Technology in Health, CPA = Canadian Psychiatry Association, PAP = Psychopharmacology Algorithm Project, PORT = Patient Outcomes Research Team, TMPA = Texas Medication Algorithm Project.

### Clozapine prescribing trends

Treatment-resistant schizophrenia has been established to affect 20-30% of all patients diagnosed with schizophrenia [21,31]. Nevertheless, physicians’ prescribing practices reveal that only a small portion of patients with treatment-resistant schizophrenia are treated with clozapine [21]. In 1999, only 160,000 US patients of the 2.6 million diagnosed with schizophrenia were treated with clozapine (only 25% of treatment-resistant patients) [21]. Since the introduction of the newer SGAs, clozapine use decreased in the United States: in 1999, clozapine represented 11% out of the total SGAs prescribed, which steadily decreased to less than 5% in 2002 [21]. During phase 3 of the CATIE study, 51% of patients had previously discontinued medications due to ineffective therapeutic response, but only 11% of patients were offered clozapine during phase 3. Studies of clozapine utilization have shown that adherence to guidelines has been poor through the years with clozapine been consistently underutilized in the US, UK, Canada, New Zealand and Australia [9,21-26,42-46]. In a study of the Veterans Health Administration, only 2% of patients affected by schizophrenia were receiving clozapine [22].

Adherence to treatment guidelines in the UK was also very poor in terms of clozapine initiation as in more than 34% of patients’ polypharmacy and high dose treatments were used before commencing clozapine [23].

Recent Canadian statistics, based on physician drug recommendations from 2005 to 2009, show a 48% increase in clozapine recommendations over a 5-year period [10]; however, a more recent report determined that 68% of an outpatient population had tried 3 or more antipsychotics before switching to clozapine [26]. In contrast, clozapine utilization in Australia appears to be more appropriate with a percentage as high as 51% of clozapine use in treatment-resistance schizophrenia [46].

## Discussion

Despite the long and still ongoing debate about the comparative safety and effectiveness of FGAs and SGAs [5,6], during the last two decades, clozapine has maintained its place in therapy as the treatment of choice in refractory schizophrenia (third-line agent). It has also been suggested that earlier use (as a second line agent) can be recommended in individuals with persistent hostility/aggressive behaviour [47] and suicidalilty [39-42]. In fact the indication for recurrent suicidal behavior has been officially approved by the FDA [48]. Its role in first-episode and non-treatment-resistant patients has been investigated [49-53] and while further research in the area of early onset schizophrenia and time to treatment response is needed [54-57], clozapine value as a second line agent has been recently recognized in a first-episode schizophrenia population [58].

While clinical practice guidelines have strongly endorsed clozapine as the gold standard of therapy, its use has been lower that recommended. Reasons for physicians’ reluctance to prescribe clozapine have been evaluated and the relatively high incidence of agranulocytosis has certainly had great influence; however, the careful monitoring of WBC counts and ANC has almost completely abolished the risk of fatal agranulocytosis; frequency of blood tests has recently been simplified and once WBC counts and ANCs have been maintained within normal levels for one year, blood tests are now only required once every 4 weeks [27,47]. It is also of importance to note that in some European countries a more flexible monitoring schedule, which can be reduced to quarterly blood testing in individuals not at risk, has been proposed [59,60].

Concerns over other adverse effects, which include weight gain, hyperglycemia, seizures, tachycardia, myocarditis and neuromalignant syndrome [61], can affect the decision making process and even less severe side effects (sedation, constipation, nocturnal hypersalivation) might result in patients’ resistance to try this medication [42,62]. Clozapine has shown, however, a definite advantage in reversing tardive dyskinesia [13,17,34,58]. Suggestions on how to balance safety and effectiveness of clozapine have been given [63]. Other issues can be seen in the added costs associated with blood monitoring and health care system contacts due to the need for gradual titration of clozapine therapy, however, clozapine cost-effectiveness has been consistently shown over other agents [64,65]. Recent reports have highlighted the need to inform health professionals about the benefits of treating patients with clozapine [24,66-69] and have voiced concern that clozapine is still under utilized especially in patients at risk of suicide [66]. Interprofessional practice models that see pharmacists being more involved in patient care have been advocated to improve and optimize prescribing of clozapine [70].

## Summary

• Published clinical evidence has supported clozapine as the gold standard in the treatment of refractory schizophrenia as it is reflected in the recommendations of many clinical practice guidelines.

• Clinical practice guidelines, however, do not appear to have been followed by prescribers and clozapine remains an underutilized medication in patients with treatment-resistant schizophrenia.

• Efforts are needed to encourage and optimize clozapine utilization.

## Abbreviations

AA: Antipsychotic agent; ANC: Absolute neutrophil count; APA: American Psychiatric Association; BAP: British Association for Psychopharmacology; BPRS: Brief Psychiatric Rating Scale; CADTH: Canadian Agency for Drugs and Technologies in Health; CPA: Canadian Psychiatry Association; CATIE: Clinical Antipsychotic Trials of Intervention Effectiveness; CUtLASS: Cost Utility of the Latest Antipsychotic Drugs in Schizophrenia Study; EPS: Extrapyramidal Symptoms; FDA: Food and Drug Administration; FGAs: First Generation Antipsychotics; NICE: National Institute of Health and Clinical Excellence; PAP: Psychopharmacology Algorithm Project; PORT: Patient Outcomes Research Team; QoL: Quality of Life; SANS: Scale for the Assessment of Negative Symptoms; SGAs: Second Generation Antipsychotics; TD: Tardive dyskinesia; TMAP: Texas Medication Algorithm Project; WBC: White blood cells.

## Competing interests

The authors declare that they have no competing interests.

## Authors’ contributions

SW conducted the bibliographic research and drafted the article. SAS designed and supervised the project, and wrote the final draft. Both authors read and approved the final manuscript.

## Pre-publication history

The pre-publication history for this paper can be accessed here:

http://www.biomedcentral.com/1471-244X/14/102/prepub
